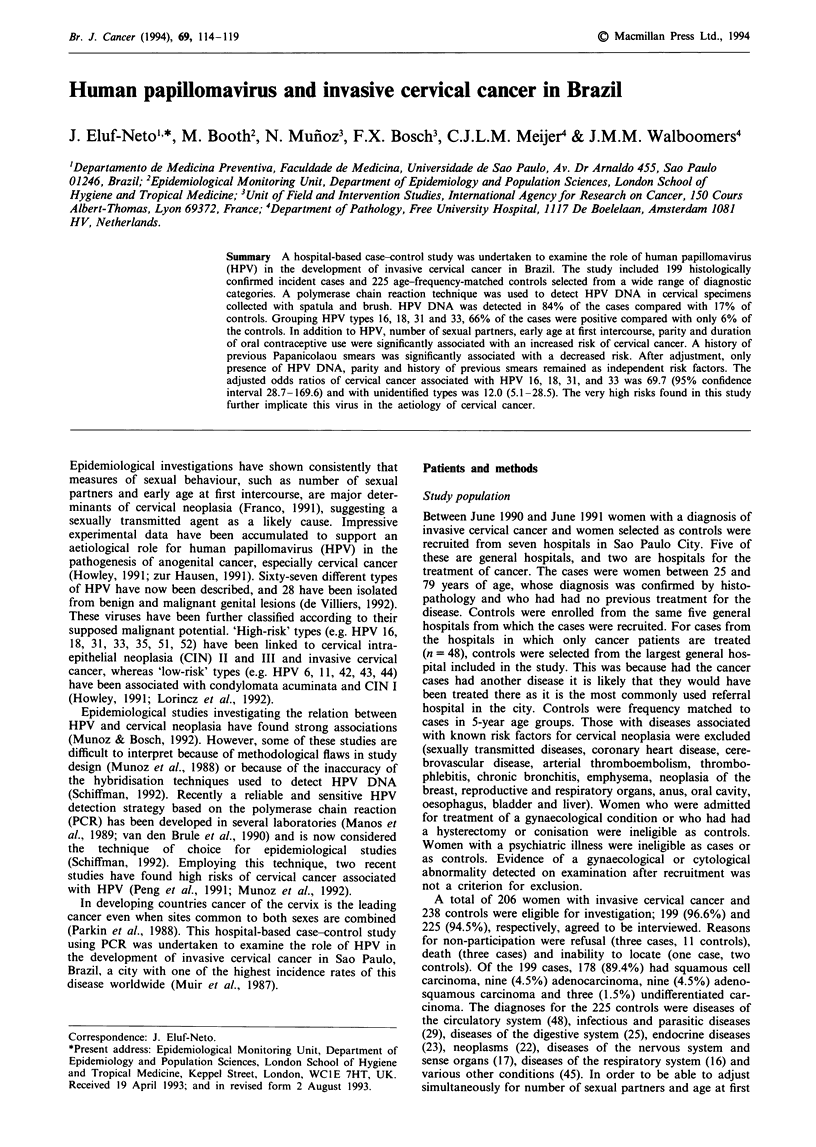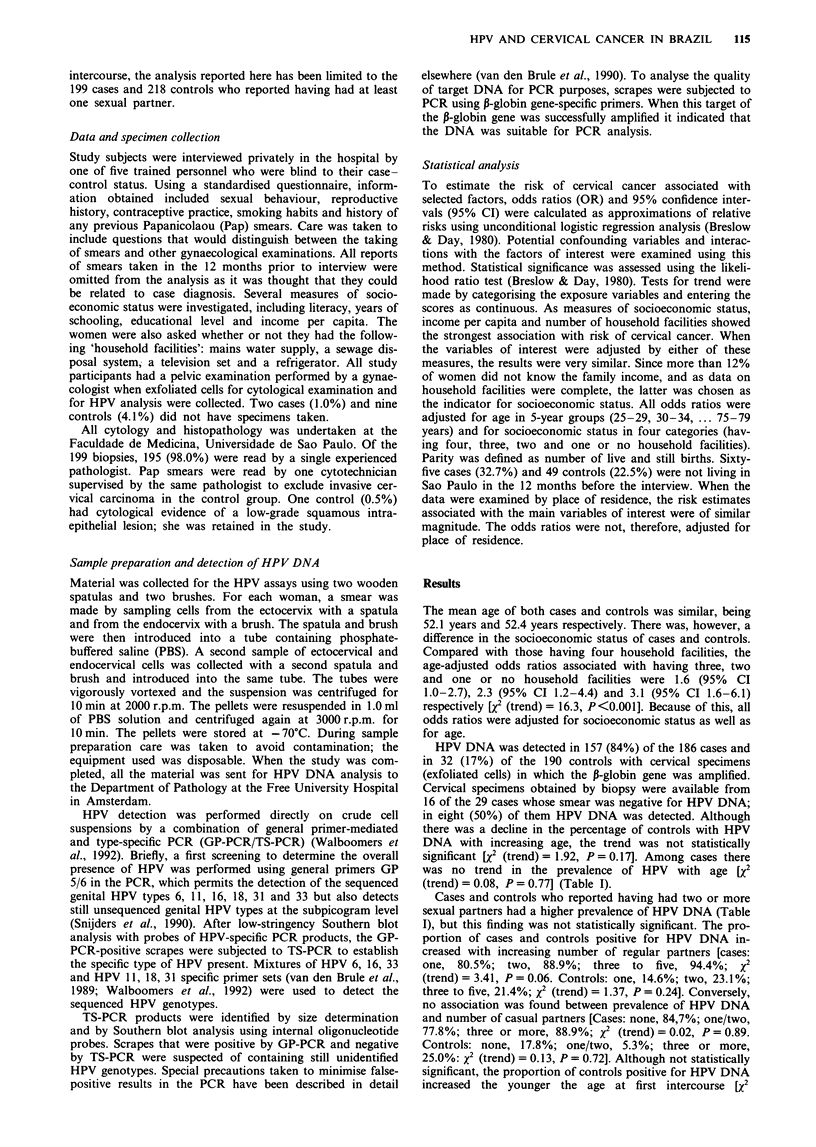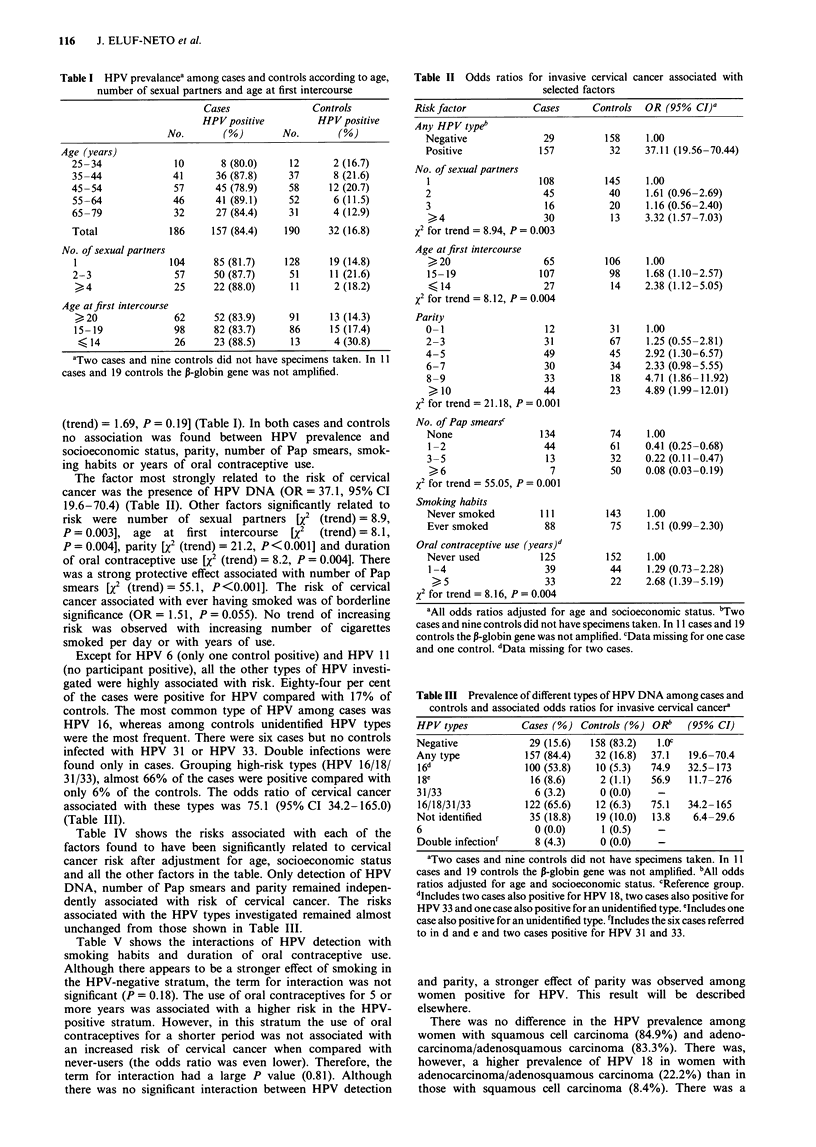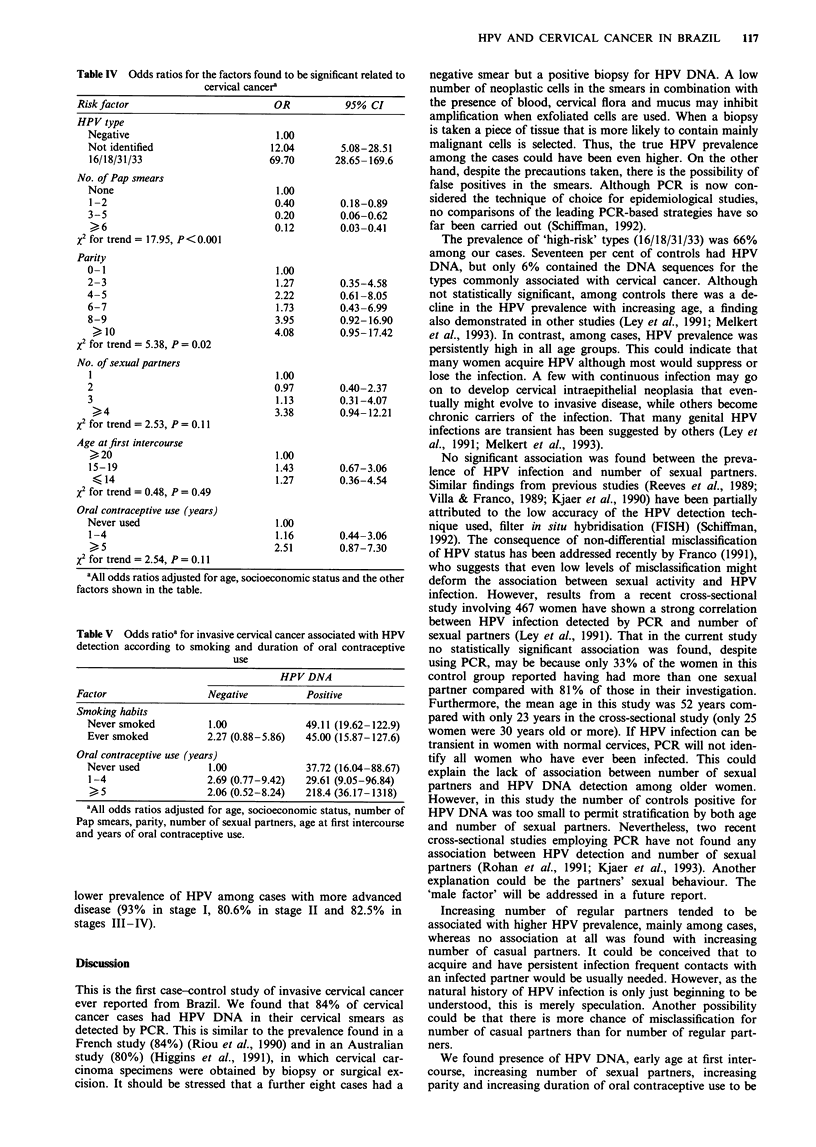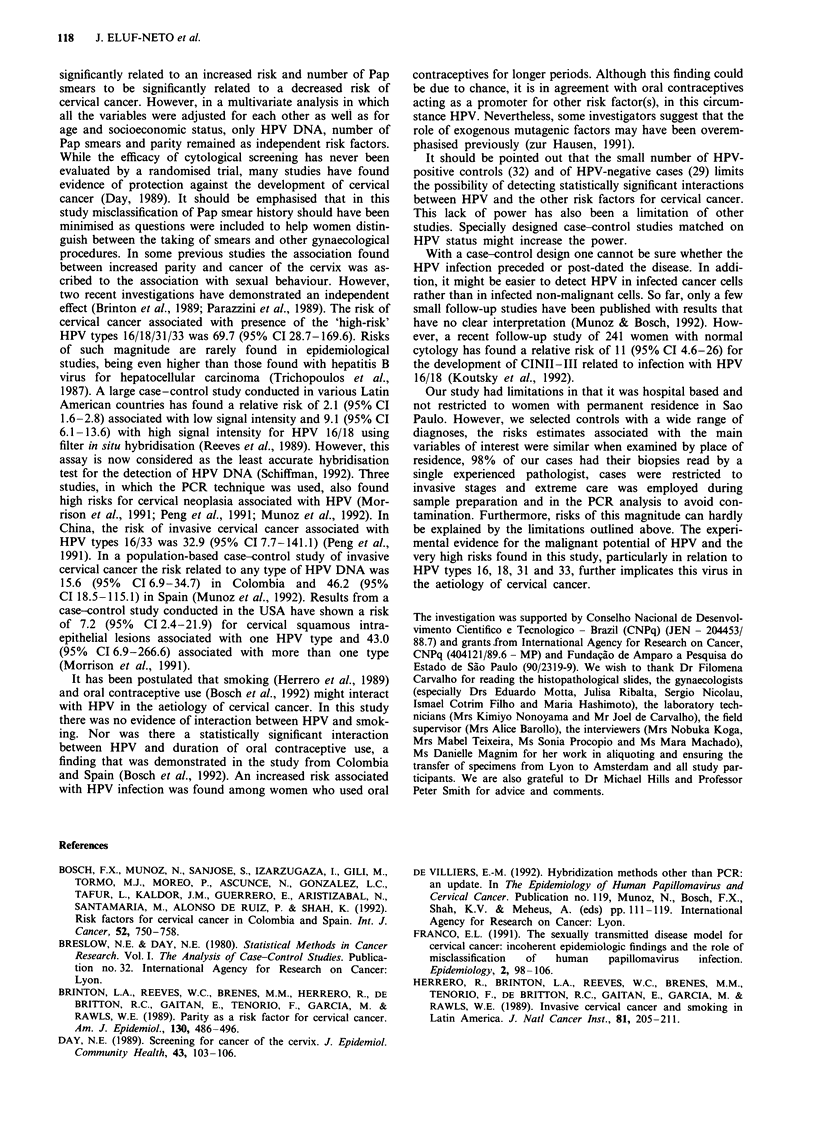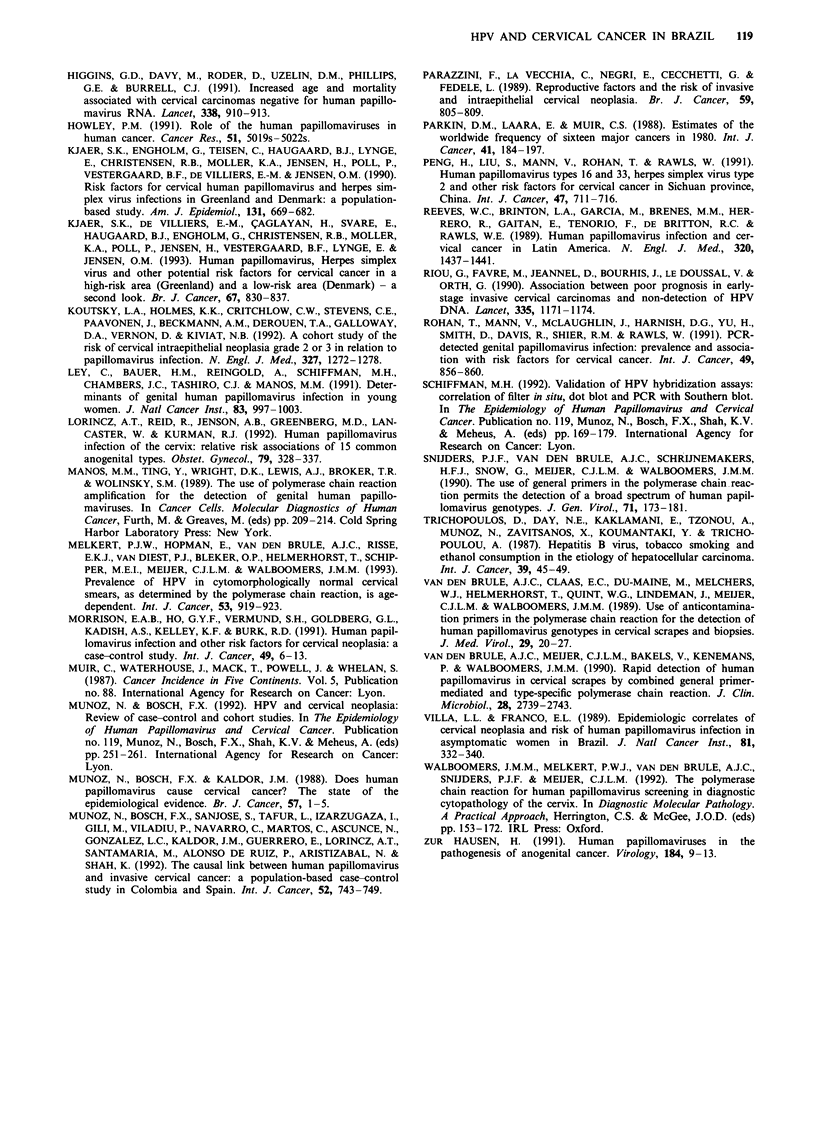# Human papillomavirus and invasive cervical cancer in Brazil.

**DOI:** 10.1038/bjc.1994.18

**Published:** 1994-01

**Authors:** J. Eluf-Neto, M. Booth, N. Muñoz, F. X. Bosch, C. J. Meijer, J. M. Walboomers

**Affiliations:** Departamento de Medicina Preventiva, Faculdade de Medicina, Universidade de Sao Paulo, Brazil.

## Abstract

A hospital-based case-control study was undertaken to examine the role of human papillomavirus (HPV) in the development of invasive cervical cancer in Brazil. The study included 199 histologically confirmed incident cases and 225 age-frequency-matched controls selected from a wide range of diagnostic categories. A polymerase chain reaction technique was used to detect HPV DNA in cervical specimens collected with spatula and brush. HPV DNA was detected in 84% of the cases compared with 17% of controls. Grouping HPV types 16, 18, 31 and 33, 66% of the cases were positive compared with only 6% of the controls. In addition to HPV, number of sexual partners, early age at first intercourse, parity and duration of oral contraceptive use were significantly associated with an increased risk of cervical cancer. A history of previous Papanicolaou smears was significantly associated with a decreased risk. After adjustment, only presence of HPV DNA, parity and history of previous smears remained as independent risk factors. The adjusted odds ratios of cervical cancer associated with HPV 16, 18, 31, and 33 was 69.7 (95% confidence interval 28.7-169.6) and with unidentified types was 12.0 (5.1-28.5). The very high risks found in this study further implicate this virus in the aetiology of cervical cancer.


Br. J. Cancer (1994), 69, 114-119 © Macmillan Press Ltd., 1994

### Human papillomavirus and invasive cervical cancer in Brazil

J. Eluf-Neto[1,*], M. Booth[2], N. Muñoz[3], F.X. Bosch[3], C.J.L.M. Meijer[4] & J.M.M. Walboomers[4]

[1]*Departamento de Medicina Preventiva, Faculdade de Medicina, Universidade de Sao Paulo, Av. Dr Arnaldo 455, Sao Paulo 01246, Brazil;* [2]*Epidemiological Monitoring Unit, Department of Epidemiology and Population Sciences, London School of Hygiene and Tropical Medicine;* [3]*Unit of Field and Intervention Studies, International Agency for Research on Cancer, 150 Cours Albert-Thomas, Lyon 69372, France;* [4]*Department of Pathology, Free University Hospital, 1117 De Boelelaan, Amsterdam 1081 HV, Netherlands.*

**Summary** A hospital-based case-control study was undertaken to examine the role of human papillomavirus (HPV) in the development of invasive cervical cancer in Brazil. The study included 199 histologically confirmed incident cases and 225 age-frequency-matched controls selected from a wide range of diagnostic categories. A polymerase chain reaction technique was used to detect HPV DNA in cervical specimens collected with spatula and brush. HPV DNA was detected in 84% of the cases compared with 17% of controls. Grouping HPV types 16, 18, 31 and 33, 66% of the cases were positive compared with only 6% of the controls. In addition to HPV, number of sexual partners, early age at first intercourse, parity and duration of oral contraceptive use were significantly associated with an increased risk of cervical cancer. A history of previous Papanicolaou smears was significantly associated with a decreased risk. After adjustment, only presence of HPV DNA, parity and history of previous smears remained as independent risk factors. The adjusted odds ratios of cervical cancer associated with HPV 16, 18, 31, and 33 was 69.7 (95% confidence interval 28.7-169.6) and with unidentified types was 12.0 (5.1-28.5). The very high risks found in this study further implicate this virus in the aetiology of cervical cancer.

Epidemiological investigations have shown consistently that measures of sexual behaviour, such as number of sexual partners and early age at first intercourse, are major determinants of cervical neoplasia (Franco, 1991), suggesting a sexually transmitted agent as a likely cause. Impressive experimental data have been accumulated to support an aetiological role for human papillomavirus (HPV) in the pathogenesis of anogenital cancer, especially cervical cancer (Howley, 1991; zur Hausen, 1991). Sixty-seven different types of HPV have now been described, and 28 have been isolated from benign and malignant genital lesions (de Villiers, 1992). These viruses have been further classified according to their supposed malignant potential. 'High-risk' types (e.g. HPV 16, 18, 31, 33, 35, 51, 52) have been linked to cervical intraepithelial neoplasia (CIN) II and III and invasive cervical cancer, whereas 'low-risk' types (e.g. HPV 6, 11, 42, 43, 44) have been associated with condylomata acuminata and CIN I (Howley, 1991; Lorincz *et al.*, 1992).

Epidemiological studies investigating the relation between HPV and cervical neoplasia have found strong associations (Munoz & Bosch, 1992). However, some of these studies are difficult to interpret because of methodological flaws in study design (Munoz *et al.*, 1988) or because of the inaccuracy of the hybridisation techniques used to detect HPV DNA (Schiffman, 1992). Recently a reliable and sensitive HPV detection strategy based on the polymerase chain reaction (PCR) has been developed in several laboratories (Manos *et al.*, 1989; van den Brule *et al.*, 1990) and is now considered the technique of choice for epidemiological studies (Schiffman, 1992). Employing this technique, two recent studies have found high risks of cervical cancer associated with HPV (Peng *et al.*, 1991; Munoz *et al.*, 1992).

In developing countries cancer of the cervix is the leading cancer even when sites common to both sexes are combined (Parkin *et al.*, 1988). This hospital-based case-control study using PCR was undertaken to examine the role of HPV in the development of invasive cervical cancer in Sao Paulo, Brazil, a city with one of the highest incidence rates of this disease worldwide (Muir *et al.*, 1987).

Correspondence: J. Eluf-Neto.

*Present address: Epidemiological Monitoring Unit, Department of Epidemiology and Population Sciences, London School of Hygiene and Tropical Medicine, Keppel Street, London, WC1E 7HT, UK.

Received 19 April 1993; and in revised form 2 August 1993.

### Patients and methods

### *Study population*

Between June 1990 and June 1991 women with a diagnosis of invasive cervical cancer and women selected as controls were recruited from seven hospitals in Sao Paulo City. Five of these are general hospitals, and two are hospitals for the treatment of cancer. The cases were women between 25 and 79 years of age, whose diagnosis was confirmed by histopathology and who had had no previous treatment for the disease. Controls were enrolled from the same five general hospitals from which the cases were recruited. For cases from the hospitals in which only cancer patients are treated ($n = 48$), controls were selected from the largest general hospital included in the study. This was because had the cancer cases had another disease it is likely that they would have been treated there as it is the most commonly used referral hospital in the city. Controls were frequency matched to cases in 5-year age groups. Those with diseases associated with known risk factors for cervical neoplasia were excluded (sexually transmitted diseases, coronary heart disease, cerebrovascular disease, arterial thromboembolism, thrombophlebitis, chronic bronchitis, emphysema, neoplasia of the breast, reproductive and respiratory organs, anus, oral cavity, oesophagus, bladder and liver). Women who were admitted for treatment of a gynaecological condition or who had had a hysterectomy or conisation were ineligible as controls. Women with a psychiatric illness were ineligible as cases or as controls. Evidence of a gynaecological or cytological abnormality detected on examination after recruitment was not a criterion for exclusion.

A total of 206 women with invasive cervical cancer and 238 controls were eligible for investigation; 199 (96.6%) and 225 (94.5%), respectively, agreed to be interviewed. Reasons for non-participation were refusal (three cases, 11 controls), death (three cases) and inability to locate (one case, two controls). Of the 199 cases, 178 (89.4%) had squamous cell carcinoma, nine (4.5%) adenocarcinoma, nine (4.5%) adenosquamous carcinoma and three (1.5%) undifferentiated carcinoma. The diagnoses for the 225 controls were diseases of the circulatory system (48), infectious and parasitic diseases (29), diseases of the digestive system (25), endocrine diseases (23), neoplasms (22), diseases of the nervous system and sense organs (17), diseases of the respiratory system (16) and various other conditions (45). In order to be able to adjust simultaneously for number of sexual partners and age at first intercourse, the analysis reported here has been limited to the 199 cases and 218 controls who reported having had at least one sexual partner.

*Data and specimen collection*

Study subjects were interviewed privately in the hospital by one of five trained personnel who were blind to their case–control status. Using a standardised questionnaire, information obtained included sexual behaviour, reproductive history, contraceptive practice, smoking habits and history of any previous Papanicolaou (Pap) smears. Care was taken to include questions that would distinguish between the taking of smears and other gynaecological examinations. All reports of smears taken in the 12 months prior to interview were omitted from the analysis as it was thought that they could be related to case diagnosis. Several measures of socioeconomic status were investigated, including literacy, years of schooling, educational level and income per capita. The women were also asked whether or not they had the following 'household facilities': mains water supply, a sewage disposal system, a television set and a refrigerator. All study participants had a pelvic examination performed by a gynaecologist when exfoliated cells for cytological examination and for HPV analysis were collected. Two cases (1.0%) and nine controls (4.1%) did not have specimens taken.

All cytology and histopathology was undertaken at the Faculdade de Medicina, Universidade de Sao Paulo. Of the 199 biopsies, 195 (98.0%) were read by a single experienced pathologist. Pap smears were read by one cytotechnician supervised by the same pathologist to exclude invasive cervical carcinoma in the control group. One control (0.5%) had cytological evidence of a low-grade squamous intraepithelial lesion; she was retained in the study.

*Sample preparation and detection of HPV DNA*

Material was collected for the HPV assays using two wooden spatulas and two brushes. For each woman, a smear was made by sampling cells from the ectocervix with a spatula and from the endocervix with a brush. The spatula and brush were then introduced into a tube containing phosphate-buffered saline (PBS). A second sample of ectocervical and endocervical cells was collected with a second spatula and brush and introduced into the same tube. The tubes were vigorously vortexed and the suspension was centrifuged for 10 min at 2000 r.p.m. The pellets were resuspended in 1.0 ml of PBS solution and centrifuged again at 3000 r.p.m. for 10 min. The pellets were stored at −70°C. During sample preparation care was taken to avoid contamination; the equipment used was disposable. When the study was completed, all the material was sent for HPV DNA analysis to the Department of Pathology at the Free University Hospital in Amsterdam.

HPV detection was performed directly on crude cell suspensions by a combination of general primer-mediated and type-specific PCR (GP-PCR/TS-PCR) (Walboomers *et al.*, 1992). Briefly, a first screening to determine the overall presence of HPV was performed using general primers GP 5/6 in the PCR, which permits the detection of the sequenced genital HPV types 6, 11, 16, 18, 31 and 33 but also detects still unsequenced genital HPV types at the subpicogram level (Snijders *et al.*, 1990). After low-stringency Southern blot analysis with probes of HPV-specific PCR products, the GP-PCR-positive scrapes were subjected to TS-PCR to establish the specific type of HPV present. Mixtures of HPV 6, 16, 33 and HPV 11, 18, 31 specific primer sets (van den Brule *et al.*, 1989; Walboomers *et al.*, 1992) were used to detect the sequenced HPV genotypes.

TS-PCR products were identified by size determination and by Southern blot analysis using internal oligonucleotide probes. Scrapes that were positive by GP-PCR and negative by TS-PCR were suspected of containing still unidentified HPV genotypes. Special precautions taken to minimise false-positive results in the PCR have been described in detail elsewhere (van den Brule *et al.*, 1990). To analyse the quality of target DNA for PCR purposes, scrapes were subjected to PCR using $\beta$-globin gene-specific primers. When this target of the $\beta$-globin gene was successfully amplified it indicated that the DNA was suitable for PCR analysis.

*Statistical analysis*

To estimate the risk of cervical cancer associated with selected factors, odds ratios (OR) and 95% confidence intervals (95% CI) were calculated as approximations of relative risks using unconditional logistic regression analysis (Breslow & Day, 1980). Potential confounding variables and interactions with the factors of interest were examined using this method. Statistical significance was assessed using the likelihood ratio test (Breslow & Day, 1980). Tests for trend were made by categorising the exposure variables and entering the scores as continuous. As measures of socioeconomic status, income per capita and number of household facilities showed the strongest association with risk of cervical cancer. When the variables of interest were adjusted by either of these measures, the results were very similar. Since more than 12% of women did not know the family income, and as data on household facilities were complete, the latter was chosen as the indicator for socioeconomic status. All odds ratios were adjusted for age in 5-year groups (25–29, 30–34, ... 75–79 years) and for socioeconomic status in four categories (having four, three, two and one or no household facilities). Parity was defined as number of live and still births. Sixty-five cases (32.7%) and 49 controls (22.5%) were not living in Sao Paulo in the 12 months before the interview. When the data were examined by place of residence, the risk estimates associated with the main variables of interest were of similar magnitude. The odds ratios were not, therefore, adjusted for place of residence.

### Results

The mean age of both cases and controls was similar, being 52.1 years and 52.4 years respectively. There was, however, a difference in the socioeconomic status of cases and controls. Compared with those having four household facilities, the age-adjusted odds ratios associated with having three, two and one or no household facilities were 1.6 (95% CI 1.0–2.7), 2.3 (95% CI 1.2–4.4) and 3.1 (95% CI 1.6–6.1) respectively [$\chi^2$ (trend) = 16.3, $P<0.001$]. Because of this, all odds ratios were adjusted for socioeconomic status as well as for age.

HPV DNA was detected in 157 (84%) of the 186 cases and in 32 (17%) of the 190 controls with cervical specimens (exfoliated cells) in which the $\beta$-globin gene was amplified. Cervical specimens obtained by biopsy were available from 16 of the 29 cases whose smear was negative for HPV DNA; in eight (50%) of them HPV DNA was detected. Although there was a decline in the percentage of controls with HPV DNA with increasing age, the trend was not statistically significant [$\chi^2$ (trend) = 1.92, $P = 0.17$]. Among cases there was no trend in the prevalence of HPV with age [$\chi^2$ (trend) = 0.08, $P = 0.77$] (Table I).

Cases and controls who reported having had two or more sexual partners had a higher prevalence of HPV DNA (Table I), but this finding was not statistically significant. The proportion of cases and controls positive for HPV DNA increased with increasing number of regular partners [cases: one, 80.5%; two, 88.9%; three to five, 94.4%; $\chi^2$ (trend) = 3.41, $P = 0.06$. Controls: one, 14.6%; two, 23.1%; three to five, 21.4%; $\chi^2$ (trend) = 1.37, $P = 0.24$]. Conversely, no association was found between prevalence of HPV DNA and number of casual partners [Cases: none, 84,7%; one/two, 77.8%; three or more, 88.9%; $\chi^2$ (trend) = 0.02, $P = 0.89$. Controls: none, 17.8%; one/two, 5.3%; three or more, 25.0%: $\chi^2$ (trend) = 0.13, $P = 0.72$]. Although not statistically significant, the proportion of controls positive for HPV DNA increased the younger the age at first intercourse [$\chi^2$

**Table I** HPV prevalance[a] among cases and controls according to age, number of sexual partners and age at first intercourse

| | Cases No. | Cases HPV positive (%) | Controls No. | Controls HPV positive (%) |
|---|---|---|---|---|
| *Age (years)* | | | | |
| 25–34 | 10 | 8 (80.0) | 12 | 2 (16.7) |
| 35–44 | 41 | 36 (87.8) | 37 | 8 (21.6) |
| 45–54 | 57 | 45 (78.9) | 58 | 12 (20.7) |
| 55–64 | 46 | 41 (89.1) | 52 | 6 (11.5) |
| 65–79 | 32 | 27 (84.4) | 31 | 4 (12.9) |
| Total | 186 | 157 (84.4) | 190 | 32 (16.8) |
| *No. of sexual partners* | | | | |
| 1 | 104 | 85 (81.7) | 128 | 19 (14.8) |
| 2–3 | 57 | 50 (87.7) | 51 | 11 (21.6) |
| ⩾4 | 25 | 22 (88.0) | 11 | 2 (18.2) |
| *Age at first intercourse* | | | | |
| ⩾20 | 62 | 52 (83.9) | 91 | 13 (14.3) |
| 15–19 | 98 | 82 (83.7) | 86 | 15 (17.4) |
| ⩽14 | 26 | 23 (88.5) | 13 | 4 (30.8) |

[a]Two cases and nine controls did not have specimens taken. In 11 cases and 19 controls the β-globin gene was not amplified.

(trend) = 1.69, $P = 0.19$] (Table I). In both cases and controls no association was found between HPV prevalence and socioeconomic status, parity, number of Pap smears, smoking habits or years of oral contraceptive use.

The factor most strongly related to the risk of cervical cancer was the presence of HPV DNA (OR = 37.1, 95% CI 19.6–70.4) (Table II). Other factors significantly related to risk were number of sexual partners [$\chi^2$ (trend) = 8.9, $P = 0.003$], age at first intercourse [$\chi^2$ (trend) = 8.1, $P = 0.004$], parity [$\chi^2$ (trend) = 21.2, $P < 0.001$] and duration of oral contraceptive use [$\chi^2$ (trend) = 8.2, $P = 0.004$]. There was a strong protective effect associated with number of Pap smears [$\chi^2$ (trend) = 55.1, $P < 0.001$]. The risk of cervical cancer associated with ever having smoked was of borderline significance (OR = 1.51, $P = 0.055$). No trend of increasing risk was observed with increasing number of cigarettes smoked per day or with years of use.

Except for HPV 6 (only one control positive) and HPV 11 (no participant positive), all the other types of HPV investigated were highly associated with risk. Eighty-four per cent of the cases were positive for HPV compared with 17% of controls. The most common type of HPV among cases was HPV 16, whereas among controls unidentified HPV types were the most frequent. There were six cases but no controls infected with HPV 31 or HPV 33. Double infections were found only in cases. Grouping high-risk types (HPV 16/18/31/33), almost 66% of the cases were positive compared with only 6% of the controls. The odds ratio of cervical cancer associated with these types was 75.1 (95% CI 34.2–165.0) (Table III).

Table IV shows the risks associated with each of the factors found to have been significantly related to cervical cancer risk after adjustment for age, socioeconomic status and all the other factors in the table. Only detection of HPV DNA, number of Pap smears and parity remained independently associated with risk of cervical cancer. The risks associated with the HPV types investigated remained almost unchanged from those shown in Table III.

Table V shows the interactions of HPV detection with smoking habits and duration of oral contraceptive use. Although there appears to be a stronger effect of smoking in the HPV-negative stratum, the term for interaction was not significant ($P = 0.18$). The use of oral contraceptives for 5 or more years was associated with a higher risk in the HPV-positive stratum. However, in this stratum the use of oral contraceptives for a shorter period was not associated with an increased risk of cervical cancer when compared with never-users (the odds ratio was even lower). Therefore, the term for interaction had a large $P$ value (0.81). Although there was no significant interaction between HPV detection

**Table II** Odds ratios for invasive cervical cancer associated with selected factors

| *Risk factor* | *Cases* | *Controls* | *OR (95% CI)[a]* |
|---|---|---|---|
| *Any HPV type[b]* | | | |
| Negative | 29 | 158 | 1.00 |
| Positive | 157 | 32 | 37.11 (19.56–70.44) |
| *No. of sexual partners* | | | |
| 1 | 108 | 145 | 1.00 |
| 2 | 45 | 40 | 1.61 (0.96–2.69) |
| 3 | 16 | 20 | 1.16 (0.56–2.40) |
| ⩾4 | 30 | 13 | 3.32 (1.57–7.03) |
| $\chi^2$ for trend = 8.94, $P = 0.003$ | | | |
| *Age at first intercourse* | | | |
| ⩾20 | 65 | 106 | 1.00 |
| 15–19 | 107 | 98 | 1.68 (1.10–2.57) |
| ⩽14 | 27 | 14 | 2.38 (1.12–5.05) |
| $\chi^2$ for trend = 8.12, $P = 0.004$ | | | |
| *Parity* | | | |
| 0–1 | 12 | 31 | 1.00 |
| 2–3 | 31 | 67 | 1.25 (0.55–2.81) |
| 4–5 | 49 | 45 | 2.92 (1.30–6.57) |
| 6–7 | 30 | 34 | 2.33 (0.98–5.55) |
| 8–9 | 33 | 18 | 4.71 (1.86–11.92) |
| ⩾10 | 44 | 23 | 4.89 (1.99–12.01) |
| $\chi^2$ for trend = 21.18, $P = 0.001$ | | | |
| *No. of Pap smears[c]* | | | |
| None | 134 | 74 | 1.00 |
| 1–2 | 44 | 61 | 0.41 (0.25–0.68) |
| 3–5 | 13 | 32 | 0.22 (0.11–0.47) |
| ⩾6 | 7 | 50 | 0.08 (0.03–0.19) |
| $\chi^2$ for trend = 55.05, $P = 0.001$ | | | |
| *Smoking habits* | | | |
| Never smoked | 111 | 143 | 1.00 |
| Ever smoked | 88 | 75 | 1.51 (0.99–2.30) |
| *Oral contraceptive use (years)[d]* | | | |
| Never used | 125 | 152 | 1.00 |
| 1–4 | 39 | 44 | 1.29 (0.73–2.28) |
| ⩾5 | 33 | 22 | 2.68 (1.39–5.19) |
| $\chi^2$ for trend = 8.16, $P = 0.004$ | | | |

[a]All odds ratios adjusted for age and socioeconomic status. [b]Two cases and nine controls did not have specimens taken. In 11 cases and 19 controls the β-globin gene was not amplified. [c]Data missing for one case and one control. [d]Data missing for two cases.

**Table III** Prevalence of different types of HPV DNA among cases and controls and associated odds ratios for invasive cervical cancer[a]

| *HPV types* | *Cases (%)* | *Controls (%)* | *OR[b]* | *(95% CI)* |
|---|---|---|---|---|
| Negative | 29 (15.6) | 158 (83.2) | 1.0[c] | |
| Any type | 157 (84.4) | 32 (16.8) | 37.1 | 19.6–70.4 |
| 16[d] | 100 (53.8) | 10 (5.3) | 74.9 | 32.5–173 |
| 18[e] | 16 (8.6) | 2 (1.1) | 56.9 | 11.7–276 |
| 31/33 | 6 (3.2) | 0 (0.0) | – | |
| 16/18/31/33 | 122 (65.6) | 12 (6.3) | 75.1 | 34.2–165 |
| Not identified | 35 (18.8) | 19 (10.0) | 13.8 | 6.4–29.6 |
| 6 | 0 (0.0) | 1 (0.5) | – | |
| Double infection[f] | 8 (4.3) | 0 (0.0) | – | |

[a]Two cases and nine controls did not have specimens taken. In 11 cases and 19 controls the β-globin gene was not amplified. [b]All odds ratios adjusted for age and socioeconomic status. [c]Reference group. [d]Includes two cases also positive for HPV 18, two cases also positive for HPV 33 and one case also positive for an unidentified type. [e]Includes one case also positive for an unidentified type. [f]Includes the six cases referred to in d and e and two cases positive for HPV 31 and 33.

and parity, a stronger effect of parity was observed among women positive for HPV. This result will be described elsewhere.

There was no difference in the HPV prevalence among women with squamous cell carcinoma (84.9%) and adenocarcinoma/adenosquamous carcinoma (83.3%). There was, however, a higher prevalence of HPV 18 in women with adenocarcinoma/adenosquamous carcinoma (22.2%) than in those with squamous cell carcinoma (8.4%). There was a

**Table IV** Odds ratios for the factors found to be significant related to cervical cancer[a]

| *Risk factor* | *OR* | *95% CI* |
|---|---|---|
| *HPV type* | | |
| Negative | 1.00 | |
| Not identified | 12.04 | 5.08–28.51 |
| 16/18/31/33 | 69.70 | 28.65–169.6 |
| *No. of Pap smears* | | |
| None | 1.00 | |
| 1–2 | 0.40 | 0.18–0.89 |
| 3–5 | 0.20 | 0.06–0.62 |
| ⩾6 | 0.12 | 0.03–0.41 |
| $\chi^2$ for trend = 17.95, $P<0.001$ | | |
| *Parity* | | |
| 0–1 | 1.00 | |
| 2–3 | 1.27 | 0.35–4.58 |
| 4–5 | 2.22 | 0.61–8.05 |
| 6–7 | 1.73 | 0.43–6.99 |
| 8–9 | 3.95 | 0.92–16.90 |
| ⩾10 | 4.08 | 0.95–17.42 |
| $\chi^2$ for trend = 5.38, $P = 0.02$ | | |
| *No. of sexual partners* | | |
| 1 | 1.00 | |
| 2 | 0.97 | 0.40–2.37 |
| 3 | 1.13 | 0.31–4.07 |
| ⩾4 | 3.38 | 0.94–12.21 |
| $\chi^2$ for trend = 2.53, $P = 0.11$ | | |
| *Age at first intercourse* | | |
| ⩾20 | 1.00 | |
| 15–19 | 1.43 | 0.67–3.06 |
| ⩽14 | 1.27 | 0.36–4.54 |
| $\chi^2$ for trend = 0.48, $P = 0.49$ | | |
| *Oral contraceptive use (years)* | | |
| Never used | 1.00 | |
| 1–4 | 1.16 | 0.44–3.06 |
| ⩾5 | 2.51 | 0.87–7.30 |
| $\chi^2$ for trend = 2.54, $P = 0.11$ | | |

[a]All odds ratios adjusted for age, socioeconomic status and the other factors shown in the table.

**Table V** Odds ratio[a] for invasive cervical cancer associated with HPV detection according to smoking and duration of oral contraceptive use

| | *HPV DNA* | |
|---|---|---|
| *Factor* | *Negative* | *Positive* |
| *Smoking habits* | | |
| Never smoked | 1.00 | 49.11 (19.62–122.9) |
| Ever smoked | 2.27 (0.88–5.86) | 45.00 (15.87–127.6) |
| *Oral contraceptive use (years)* | | |
| Never used | 1.00 | 37.72 (16.04–88.67) |
| 1–4 | 2.69 (0.77–9.42) | 29.61 (9.05–96.84) |
| ⩾5 | 2.06 (0.52–8.24) | 218.4 (36.17–1318) |

[a]All odds ratios adjusted for age, socioeconomic status, number of Pap smears, parity, number of sexual partners, age at first intercourse and years of oral contraceptive use.

lower prevalence of HPV among cases with more advanced disease (93% in stage I, 80.6% in stage II and 82.5% in stages III–IV).

### Discussion

This is the first case–control study of invasive cervical cancer ever reported from Brazil. We found that 84% of cervical cancer cases had HPV DNA in their cervical smears as detected by PCR. This is similar to the prevalence found in a French study (84%) (Riou *et al.*, 1990) and in an Australian study (80%) (Higgins *et al.*, 1991), in which cervical carcinoma specimens were obtained by biopsy or surgical excision. It should be stressed that a further eight cases had a negative smear but a positive biopsy for HPV DNA. A low number of neoplastic cells in the smears in combination with the presence of blood, cervical flora and mucus may inhibit amplification when exfoliated cells are used. When a biopsy is taken a piece of tissue that is more likely to contain mainly malignant cells is selected. Thus, the true HPV prevalence among the cases could have been even higher. On the other hand, despite the precautions taken, there is the possibility of false positives in the smears. Although PCR is now considered the technique of choice for epidemiological studies, no comparisons of the leading PCR-based strategies have so far been carried out (Schiffman, 1992).

The prevalence of 'high-risk' types (16/18/31/33) was 66% among our cases. Seventeen per cent of controls had HPV DNA, but only 6% contained the DNA sequences for the types commonly associated with cervical cancer. Although not statistically significant, among controls there was a decline in the HPV prevalence with increasing age, a finding also demonstrated in other studies (Ley *et al.*, 1991; Melkert *et al.*, 1993). In contrast, among cases, HPV prevalence was persistently high in all age groups. This could indicate that many women acquire HPV although most would suppress or lose the infection. A few with continuous infection may go on to develop cervical intraepithelial neoplasia that eventually might evolve to invasive disease, while others become chronic carriers of the infection. That many genital HPV infections are transient has been suggested by others (Ley *et al.*, 1991; Melkert *et al.*, 1993).

No significant association was found between the prevalence of HPV infection and number of sexual partners. Similar findings from previous studies (Reeves *et al.*, 1989; Villa & Franco, 1989; Kjaer *et al.*, 1990) have been partially attributed to the low accuracy of the HPV detection technique used, filter *in situ* hybridisation (FISH) (Schiffman, 1992). The consequence of non-differential misclassification of HPV status has been addressed recently by Franco (1991), who suggests that even low levels of misclassification might deform the association between sexual activity and HPV infection. However, results from a recent cross-sectional study involving 467 women have shown a strong correlation between HPV infection detected by PCR and number of sexual partners (Ley *et al.*, 1991). That in the current study no statistically significant association was found, despite using PCR, may be because only 33% of the women in this control group reported having had more than one sexual partner compared with 81% of those in their investigation. Furthermore, the mean age in this study was 52 years compared with only 23 years in the cross-sectional study (only 25 women were 30 years old or more). If HPV infection can be transient in women with normal cervices, PCR will not identify all women who have ever been infected. This could explain the lack of association between number of sexual partners and HPV DNA detection among older women. However, in this study the number of controls positive for HPV DNA was too small to permit stratification by both age and number of sexual partners. Nevertheless, two recent cross-sectional studies employing PCR have not found any association between HPV detection and number of sexual partners (Rohan *et al.*, 1991; Kjaer *et al.*, 1993). Another explanation could be the partners' sexual behaviour. The 'male factor' will be addressed in a future report.

Increasing number of regular partners tended to be associated with higher HPV prevalence, mainly among cases, whereas no association at all was found with increasing number of casual partners. It could be conceived that to acquire and have persistent infection frequent contacts with an infected partner would be usually needed. However, as the natural history of HPV infection is only just beginning to be understood, this is merely speculation. Another possibility could be that there is more chance of misclassification for number of casual partners than for number of regular partners.

We found presence of HPV DNA, early age at first intercourse, increasing number of sexual partners, increasing parity and increasing duration of oral contraceptive use to be significantly related to an increased risk and number of Pap smears to be significantly related to a decreased risk of cervical cancer. However, in a multivariate analysis in which all the variables were adjusted for each other as well as for age and socioeconomic status, only HPV DNA, number of Pap smears and parity remained as independent risk factors. While the efficacy of cytological screening has never been evaluated by a randomised trial, many studies have found evidence of protection against the development of cervical cancer (Day, 1989). It should be emphasised that in this study misclassification of Pap smear history should have been minimised as questions were included to help women distinguish between the taking of smears and other gynaecological procedures. In some previous studies the association found between increased parity and cancer of the cervix was ascribed to the association with sexual behaviour. However, two recent investigations have demonstrated an independent effect (Brinton *et al.*, 1989; Parazzini *et al.*, 1989). The risk of cervical cancer associated with presence of the 'high-risk' HPV types 16/18/31/33 was 69.7 (95% CI 28.7–169.6). Risks of such magnitude are rarely found in epidemiological studies, being even higher than those found with hepatitis B virus for hepatocellular carcinoma (Trichopoulos *et al.*, 1987). A large case–control study conducted in various Latin American countries has found a relative risk of 2.1 (95% CI 1.6–2.8) associated with low signal intensity and 9.1 (95% CI 6.1–13.6) with high signal intensity for HPV 16/18 using filter *in situ* hybridisation (Reeves *et al.*, 1989). However, this assay is now considered as the least accurate hybridisation test for the detection of HPV DNA (Schiffman, 1992). Three studies, in which the PCR technique was used, also found high risks for cervical neoplasia associated with HPV (Morrison *et al.*, 1991; Peng *et al.*, 1991; Munoz *et al.*, 1992). In China, the risk of invasive cervical cancer associated with HPV types 16/33 was 32.9 (95% CI 7.7–141.1) (Peng *et al.*, 1991). In a population-based case–control study of invasive cervical cancer the risk related to any type of HPV DNA was 15.6 (95% CI 6.9–34.7) in Colombia and 46.2 (95% CI 18.5–115.1) in Spain (Munoz *et al.*, 1992). Results from a case–control study conducted in the USA have shown a risk of 7.2 (95% CI 2.4–21.9) for cervical squamous intraepithelial lesions associated with one HPV type and 43.0 (95% CI 6.9–266.6) associated with more than one type (Morrison *et al.*, 1991).

It has been postulated that smoking (Herrero *et al.*, 1989) and oral contraceptive use (Bosch *et al.*, 1992) might interact with HPV in the aetiology of cervical cancer. In this study there was no evidence of interaction between HPV and smoking. Nor was there a statistically significant interaction between HPV and duration of oral contraceptive use, a finding that was demonstrated in the study from Colombia and Spain (Bosch *et al.*, 1992). An increased risk associated with HPV infection was found among women who used oral contraceptives for longer periods. Although this finding could be due to chance, it is in agreement with oral contraceptives acting as a promoter for other risk factor(s), in this circumstance HPV. Nevertheless, some investigators suggest that the role of exogenous mutagenic factors may have been overemphasised previously (zur Hausen, 1991).

It should be pointed out that the small number of HPV-positive controls (32) and of HPV-negative cases (29) limits the possibility of detecting statistically significant interactions between HPV and the other risk factors for cervical cancer. This lack of power has also been a limitation of other studies. Specially designed case–control studies matched on HPV status might increase the power.

With a case–control design one cannot be sure whether the HPV infection preceded or post-dated the disease. In addition, it might be easier to detect HPV in infected cancer cells rather than in infected non-malignant cells. So far, only a few small follow-up studies have been published with results that have no clear interpretation (Munoz & Bosch, 1992). However, a recent follow-up study of 241 women with normal cytology has found a relative risk of 11 (95% CI 4.6–26) for the development of CINII–III related to infection with HPV 16/18 (Koutsky *et al.*, 1992).

Our study had limitations in that it was hospital based and not restricted to women with permanent residence in Sao Paulo. However, we selected controls with a wide range of diagnoses, the risks estimates associated with the main variables of interest were similar when examined by place of residence, 98% of our cases had their biopsies read by a single experienced pathologist, cases were restricted to invasive stages and extreme care was employed during sample preparation and in the PCR analysis to avoid contamination. Furthermore, risks of this magnitude can hardly be explained by the limitations outlined above. The experimental evidence for the malignant potential of HPV and the very high risks found in this study, particularly in relation to HPV types 16, 18, 31 and 33, further implicates this virus in the aetiology of cervical cancer.

The investigation was supported by Conselho Nacional de Desenvolvimento Cientifico e Tecnologico - Brazil (CNPq) (JEN - 204453/88.7) and grants from International Agency for Research on Cancer, CNPq (404121/89.6 - MP) and Fundação de Amparo a Pesquisa do Estado de São Paulo (90/2319-9). We wish to thank Dr Filomena Carvalho for reading the histopathological slides, the gynaecologists (especially Drs Eduardo Motta, Julisa Ribalta, Sergio Nicolau, Ismael Cotrim Filho and Maria Hashimoto), the laboratory technicians (Mrs Kimiyo Nonoyama and Mr Joel de Carvalho), the field supervisor (Mrs Alice Barollo), the interviewers (Mrs Nobuka Koga, Mrs Mabel Teixeira, Ms Sonia Procopio and Ms Mara Machado), Ms Danielle Magnim for her work in aliquoting and ensuring the transfer of specimens from Lyon to Amsterdam and all study participants. We are also grateful to Dr Michael Hills and Professor Peter Smith for advice and comments.

### References

BOSCH, F.X., MUNOZ, N., SANJOSE, S., IZARZUGAZA, I., GILI, M., TORMO, M.J., MOREO, P., ASCUNCE, N., GONZALEZ, L.C., TAFUR, L., KALDOR, J.M., GUERRERO, E., ARISTIZABAL, N., SANTAMARIA, M., ALONSO DE RUIZ, P. & SHAH, K. (1992). Risk factors for cervical cancer in Colombia and Spain. *Int. J. Cancer*, **52**, 750–758.

BRESLOW, N.E. & DAY, N.E. (1980). *Statistical Methods in Cancer Research*. Vol. I. *The Analysis of Case-Control Studies*. Publication no. 32. International Agency for Research on Cancer: Lyon.

BRINTON, L.A., REEVES, W.C., BRENES, M.M., HERRERO, R., DE BRITTON, R.C., GAITAN, E., TENORIO, F., GARCIA, M. & RAWLS, W.E. (1989). Parity as a risk factor for cervical cancer. *Am. J. Epidemiol.*, **130**, 486–496.

DAY, N.E. (1989). Screening for cancer of the cervix. *J. Epidemiol. Community Health*, **43**, 103–106.

DE VILLIERS, E.-M. (1992). Hybridization methods other than PCR: an update. In *The Epidemiology of Human Papillomavirus and Cervical Cancer*. Publication no. 119, Munoz, N., Bosch, F.X., Shah, K.V. & Meheus, A. (eds) pp. 111–119. International Agency for Research on Cancer: Lyon.

FRANCO, E.L. (1991). The sexually transmitted disease model for cervical cancer: incoherent epidemiologic findings and the role of misclassification of human papillomavirus infection. *Epidemiology*, **2**, 98–106.

HERRERO, R., BRINTON, L.A., REEVES, W.C., BRENES, M.M., TENORIO, F., DE BRITTON, R.C., GAITAN, E., GARCIA, M. & RAWLS, W.E. (1989). Invasive cervical cancer and smoking in Latin America. *J. Natl Cancer Inst.*, **81**, 205–211.

HIGGINS, G.D., DAVY, M., RODER, D., UZELIN, D.M., PHILLIPS, G.E. & BURRELL, C.J. (1991). Increased age and mortality associated with cervical carcinomas negative for human papillomavirus RNA. *Lancet*, **338**, 910–913.

HOWLEY, P.M. (1991). Role of the human papillomaviruses in human cancer. *Cancer Res.*, **51**, 5019s–5022s.

KJAER, S.K., ENGHOLM, G., TEISEN, C., HAUGAARD, B.J., LYNGE, E., CHRISTENSEN, R.B., MOLLER, K.A., JENSEN, H., POLL, P., VESTERGAARD, B.F., DE VILLIERS, E.-M. & JENSEN, O.M. (1990). Risk factors for cervical human papillomavirus and herpes simplex virus infections in Greenland and Denmark: a population-based study. *Am. J. Epidemiol.*, **131**, 669–682.

KJAER, S.K., DE VILLIERS, E.-M., ÇAGLAYAN, H., SVARE, E., HAUGAARD, B.J., ENGHOLM, G., CHRISTENSEN, R.B., MOLLER, K.A., POLL, P., JENSEN, H., VESTERGAARD, B.F., LYNGE, E. & JENSEN, O.M. (1993). Human papillomavirus, Herpes simplex virus and other potential risk factors for cervical cancer in a high-risk area (Greenland) and a low-risk area (Denmark) – a second look. *Br. J. Cancer*, **67**, 830–837.

KOUTSKY, L.A., HOLMES, K.K., CRITCHLOW, C.W., STEVENS, C.E., PAAVONEN, J., BECKMANN, A.M., DEROUEN, T.A., GALLOWAY, D.A., VERNON, D. & KIVIAT, N.B. (1992). A cohort study of the risk of cervical intraepithelial neoplasia grade 2 or 3 in relation to papillomavirus infection. *N. Engl. J. Med.*, **327**, 1272–1278.

LEY, C., BAUER, H.M., REINGOLD, A., SCHIFFMAN, M.H., CHAMBERS, J.C., TASHIRO, C.J. & MANOS, M.M. (1991). Determinants of genital human papillomavirus infection in young women. *J. Natl Cancer Inst.*, **83**, 997–1003.

LORINCZ, A.T., REID, R., JENSON, A.B., GREENBERG, M.D., LANCASTER, W. & KURMAN, R.J. (1992). Human papillomavirus infection of the cervix: relative risk associations of 15 common anogenital types. *Obstet. Gynecol.*, **79**, 328–337.

MANOS, M.M., TING, Y., WRIGHT, D.K., LEWIS, A.J., BROKER, T.R. & WOLINSKY, S.M. (1989). The use of polymerase chain reaction amplification for the detection of genital human papillomaviruses. In *Cancer Cells. Molecular Diagnostics of Human Cancer*, Furth, M. & Greaves, M. (eds) pp. 209–214. Cold Spring Harbor Laboratory Press: New York.

MELKERT, P.J.W., HOPMAN, E., VAN DEN BRULE, A.J.C., RISSE, E.K.J., VAN DIEST, P.J., BLEKER, O.P., HELMERHORST, T., SCHIPPER, M.E.I., MEIJER, C.J.L.M. & WALBOOMERS, J.M.M. (1993). Prevalence of HPV in cytomorphologically normal cervical smears, as determined by the polymerase chain reaction, is age-dependent. *Int. J. Cancer*, **53**, 919–923.

MORRISON, E.A.B., HO, G.Y.F., VERMUND, S.H., GOLDBERG, G.L., KADISH, A.S., KELLEY, K.F. & BURK, R.D. (1991). Human papillomavirus infection and other risk factors for cervical neoplasia: a case–control study. *Int. J. Cancer*, **49**, 6–13.

MUIR, C., WATERHOUSE, J., MACK, T., POWELL, J. & WHELAN, S. (1987). *Cancer Incidence in Five Continents*. Vol. 5, Publication no. 88. International Agency for Research on Cancer: Lyon.

MUNOZ, N. & BOSCH, F.X. (1992). HPV and cervical neoplasia: Review of case–control and cohort studies. In *The Epidemiology of Human Papillomavirus and Cervical Cancer*. Publication no. 119, Munoz, N., Bosch, F.X., Shah, K.V. & Meheus, A. (eds) pp. 251–261. International Agency for Research on Cancer: Lyon.

MUNOZ, N., BOSCH, F.X. & KALDOR, J.M. (1988). Does human papillomavirus cause cervical cancer? The state of the epidemiological evidence. *Br. J. Cancer*, **57**, 1–5.

MUNOZ, N., BOSCH, F.X., SANJOSE, S., TAFUR, L., IZARZUGAZA, I., GILI, M., VILADIU, P., NAVARRO, C., MARTOS, C., ASCUNCE, N., GONZALEZ, L.C., KALDOR, J.M., GUERRERO, E., LORINCZ, A.T., SANTAMARIA, M., ALONSO DE RUIZ, P., ARISTIZABAL, N. & SHAH, K. (1992). The causal link between human papillomavirus and invasive cervical cancer: a population-based case–control study in Colombia and Spain. *Int. J. Cancer*, **52**, 743–749.

PARAZZINI, F., LA VECCHIA, C., NEGRI, E., CECCHETTI, G. & FEDELE, L. (1989). Reproductive factors and the risk of invasive and intraepithelial cervical neoplasia. *Br. J. Cancer*, **59**, 805–809.

PARKIN, D.M., LAARA, E. & MUIR, C.S. (1988). Estimates of the worldwide frequency of sixteen major cancers in 1980. *Int. J. Cancer*, **41**, 184–197.

PENG, H., LIU, S., MANN, V., ROHAN, T. & RAWLS, W. (1991). Human papillomavirus types 16 and 33, herpes simplex virus type 2 and other risk factors for cervical cancer in Sichuan province, China. *Int. J. Cancer*, **47**, 711–716.

REEVES, W.C., BRINTON, L.A., GARCIA, M., BRENES, M.M., HERRERO, R., GAITAN, E., TENORIO, F., DE BRITTON, R.C. & RAWLS, W.E. (1989). Human papillomavirus infection and cervical cancer in Latin America. *N. Engl. J. Med.*, **320**, 1437–1441.

RIOU, G., FAVRE, M., JEANNEL, D., BOURHIS, J., LE DOUSSAL, V. & ORTH, G. (1990). Association between poor prognosis in early-stage invasive cervical carcinomas and non-detection of HPV DNA. *Lancet*, **335**, 1171–1174.

ROHAN, T., MANN, V., MCLAUGHLIN, J., HARNISH, D.G., YU, H., SMITH, D., DAVIS, R., SHIER, R.M. & RAWLS, W. (1991). PCR-detected genital papillomavirus infection: prevalence and association with risk factors for cervical cancer. *Int. J. Cancer*, **49**, 856–860.

SCHIFFMAN, M.H. (1992). Validation of HPV hybridization assays: correlation of filter *in situ*, dot blot and PCR with Southern blot. In *The Epidemiology of Human Papillomavirus and Cervical Cancer*. Publication no. 119, Munoz, N., Bosch, F.X., Shah, K.V. & Meheus, A. (eds) pp. 169–179. International Agency for Research on Cancer: Lyon.

SNIJDERS, P.J.F., VAN DEN BRULE, A.J.C., SCHRIJNEMAKERS, H.F.J., SNOW, G., MEIJER, C.J.L.M. & WALBOOMERS, J.M.M. (1990). The use of general primers in the polymerase chain reaction permits the detection of a broad spectrum of human papillomavirus genotypes. *J. Gen. Virol.*, **71**, 173–181.

TRICHOPOULOS, D., DAY, N.E., KAKLAMANI, E., TZONOU, A., MUNOZ, N., ZAVITSANOS, X., KOUMANTAKI, Y. & TRICHOPOULOU, A. (1987). Hepatitis B virus, tobacco smoking and ethanol consumption in the etiology of hepatocellular carcinoma. *Int. J. Cancer*, **39**, 45–49.

VAN DEN BRULE, A.J.C., CLAAS, E.C., DU-MAINE, M., MELCHERS, W.J., HELMERHORST, T., QUINT, W.G., LINDEMAN, J., MEIJER, C.J.L.M. & WALBOOMERS, J.M.M. (1989). Use of anticontamination primers in the polymerase chain reaction for the detection of human papillomavirus genotypes in cervical scrapes and biopsies. *J. Med. Virol.*, **29**, 20–27.

VAN DEN BRULE, A.J.C., MEIJER, C.J.L.M., BAKELS, V., KENEMANS, P. & WALBOOMERS, J.M.M. (1990). Rapid detection of human papillomavirus in cervical scrapes by combined general primer-mediated and type-specific polymerase chain reaction. *J. Clin. Microbiol.*, **28**, 2739–2743.

VILLA, L.L. & FRANCO, E.L. (1989). Epidemiologic correlates of cervical neoplasia and risk of human papillomavirus infection in asymptomatic women in Brazil. *J. Natl Cancer Inst.*, **81**, 332–340.

WALBOOMERS, J.M.M., MELKERT, P.W.J., VAN DEN BRULE, A.J.C., SNIJDERS, P.J.F. & MEIJER, C.J.L.M. (1992). The polymerase chain reaction for human papillomavirus screening in diagnostic cytopathology of the cervix. In *Diagnostic Molecular Pathology. A Practical Approach*, Herrington, C.S. & McGee, J.O.D. (eds) pp. 153–172. IRL Press: Oxford.

ZUR HAUSEN, H. (1991). Human papillomaviruses in the pathogenesis of anogenital cancer. *Virology*, **184**, 9–13.